# Indole Derivatives Produced by the Metagenome Genes of the *Escherichia coli*-Harboring Marine Sponge *Discodermia calyx*

**DOI:** 10.3390/molecules22050681

**Published:** 2017-04-25

**Authors:** Feng-Lou Liu, Xiao-Long Yang

**Affiliations:** 1 Agricultural college, Ningxia University, Yinchuan 750021, Ningxia, China; liufenglou@nxu.edu.cn; 2 Innovative Drug Research Centre (IDRC), School of Pharmaceutical Sciences, Huxi Campus, Chongqing University, Chongqing 401331, China

**Keywords:** *Discodermia calyx*, indole derivative, antibacterial activity, metagenomics

## Abstract

Three indole derivatives, a novel benzoxazine-indole hybrid (**1**) and two known indole trimers (**2**, **3**), were isolated from the metagenomic library of the marine sponge *Discodermia calyx* based on functional screening. Their structures were elucidated by extensive spectroscopic analysis and comparison of their NMR data to that of known compounds. The antibacterial assay indicated that only compound **2** displayed significant antibacterial activity against *Bacillus cereus*, with approximately 20 mm diameter growth inhibition at 10 µg/paper. HPLC analyses revealed that compound **2** is a newly induced metabolite, and the concentration of **3** was obviously enhanced in contrast to negative control, while **1** was not detected, allowing us to predict that the formation of **2** might be induced by exogenous genes derived from the sponge metagenome, whereas compound **1** could be formed through a non-enzymatic process during the isolation procedure.

## 1. Introduction

Marine sponges and their associated microorganisms have been attracting the attention of chemists and pharmacologists as a very promising potential source of novel bioactive compounds since the discovery of the nucleosides spongothymidine and spongouridine in the marine sponge *Cryptotethya crypta* in the 1950s [1]. To date, a variety of chemically interesting and biologically significant secondary metabolites have been reported from marine sponges, and most of them have shown significant antibacterial activities [2,3]. There is increasing evidence suggesting that sponge-derived symbiotic bacteria are the original producers of these bioactive compounds [4,5]. Unfortunately, only very small fraction of the total sponge-associated bacteria is amenable to cultivation and chemical study, and the vast majority of them remain uncultured, representing an enormous potential source of novel biosynthetic enzymes and secondary metabolites [5,6]. Metagenomic approaches have proven to be powerful tools for accessing the biosynthetic potential of uncultured bacteria metabolic and identifying novel metabolites by use of libraries constructed from isolated nucleic acids [7,8].

**Figure 1:**
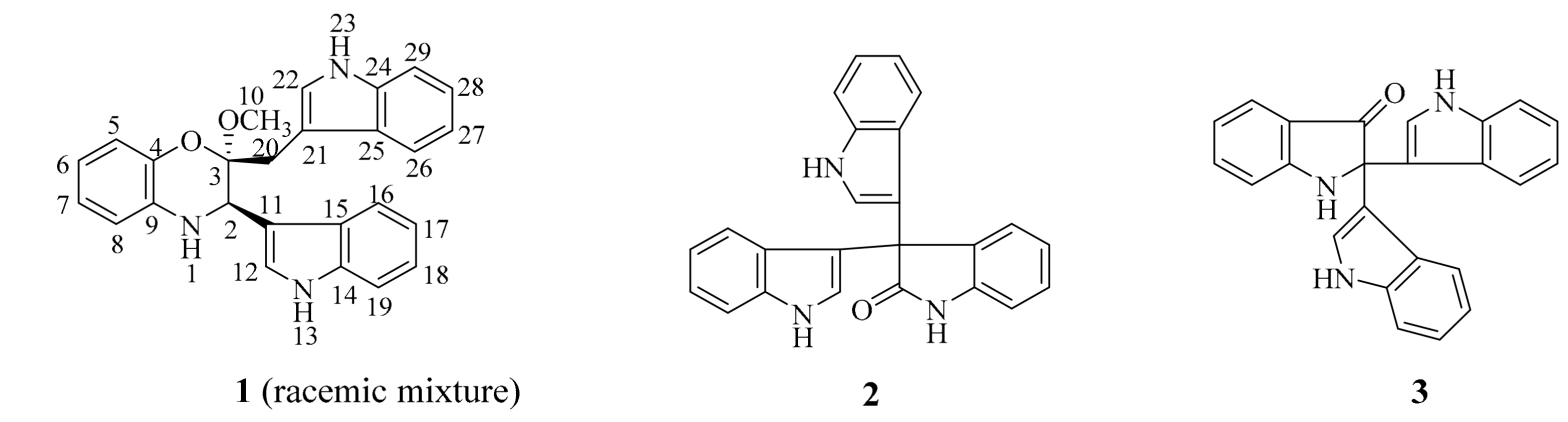
The structures of compounds **1**–**3**.

The marine sponge *Discodermia calyx* is considered one of most prolific marine producers of novel biologically active compounds, exemplified by the potent antitumor metabolites calyculins [9], and cytotoxic calyxamide cyclic peptides [10], etc. Previous investigation of the *Discodermia calyx* metagenomic library led to the isolation of porphyrins, indole antibiotics, and cyclodipeptides [11,12,13,14,15], indicating that the *D. calyx* metagenomic library is worthy of further functional screening-based investigation. Therefore, the metagenomic library, containing 2.5 × 10^5^ clones harboring ca. 35 kb of insert DNAs derived from the marine sponge *D. calyx*, was re-screened for antibacterial activity using the two-layer overlay method, resulting in the discovery of one positive clone, designated as pDC115, which produced a clear inhibition zone against *Bacillus cereus*. Further bioassay-guided fractionation yielded three indole derivatives **1**–**3**, including a novel benzoxazine-indole hybrid **1** isolated as racemic mixture (Figure 1). Herein, details of the isolation, structural elucidation and antibacterial activities of these compounds are reported.

## 2. Results and Discussion

**Figure 2:**
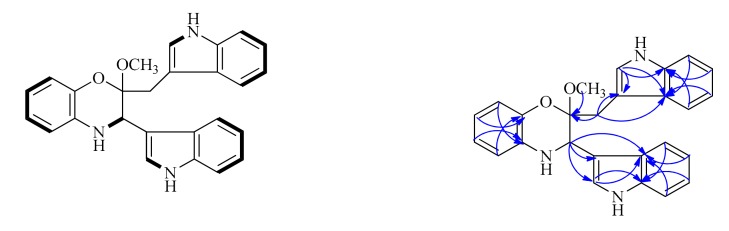
The key ^1^H, ^1^H-COSY and HMBC correlations of **1**.

Compound **1** was isolated as a colorless amorphous powder. The positive-ion ESI-TOF-MS and LC-MS showed a pseudo-molecular ion peak [M + H]^+^ at *m*/*z* 410. Its molecular formula was determined as C_26_H_23_N_3_O_2_ by the HR-ESI-MS peak at *m*/*z* 410.1887 (calcd for C_26_H_24_N_3_O_2_, *m*/*z* 410.1863), indicating 17 degrees of unsaturation. The cross-peak correlations of H-1 with H-2, H-5 with H-6, H-7 and H-8, H-12 with H-13, H-16 with H-17, H-18 and H-19, H-22 with H-23, H-26 with H-27, H-28 and H-29 observed in the ^1^H, ^1^H-COSY spectrum (Figure 2), allowed us to determine six H-atom systems as follows: from H-1 to H-2, from H-5 to H-8, from H-12 to H-13, from H-16 to H-19, from H-22 to H-23, and from H-26 to H-29. The ^1^H-NMR data (δ_H_ 10.83 (1H, s), 7.57 (1H, d), 7.30 (1H, brs), 7.02 (1H, overlap), 6.92 (1H, overlap) and 6.81 (1H, brs); 11.09 (1H, s), 7.43 (1H, d), 7.39 (1H, d), 7.30 (1H, brs), 7.07 (1H, overlap) and 6.92 (1H, overlap), Table 1), revealed the presence of two trisubstituted indole-ring moieties, which was further confirmed by analysis of the key correlations in HMBC experiments as follows: H-12 with C-14 and C-15, H-16 with C-14, H-17 with C-15, H-18 with C-14, H-19 with C-15, H-22 with C-21, C-24 and C-25, H-26 with C-24, H-27 with C-25, H-28 with C-24, H-29 with C-25. In addition, the ^1^H-NMR spectrum showed one broadened *ortho*-coupled doublet at δ_H_ 6.57 (1H, d) and one broadened singlet at δ_H_ 6.81 (1H, brs), and two triplets at δ_H_ 6.66 (1H, t), and 6.51 (1H, t), suggesting the presence of a 1,2-disubstituted benzene ring. Two indole-ring moieties along with one benzene ring accounted for 16 of 17 degrees of unsaturation, suggesting that the remaining degree of unsaturation had to be present as another ring. Careful analysis of the remaining proton signals combined with the HSQC spectrum and molecular formula revealed the presence of one methylene group (δ_H_ 2.89 (1H, d), 3.46 (1H, overlap); δ_C_ 28.7 (t)), one methoxy group (δ_H_ 3.17 (3H, s); δ_C_ 48.3 (q)), and one methine group attached to a nitrogen atom (δ_H_ 4.46 (1H, s); δ_C_ 51.2 (d)). Further detailed interpretation of the remaining HMBC correlations of H-2 with C-11, C-12 and C-15, H-10 with C-3, H-20 with C-3, C-21 and C-25, combined with the remaining single degree of unsaturation all added up to one ring, allowing us to determine its gross structure as shown in Figure 1.

**Scheme 1:**
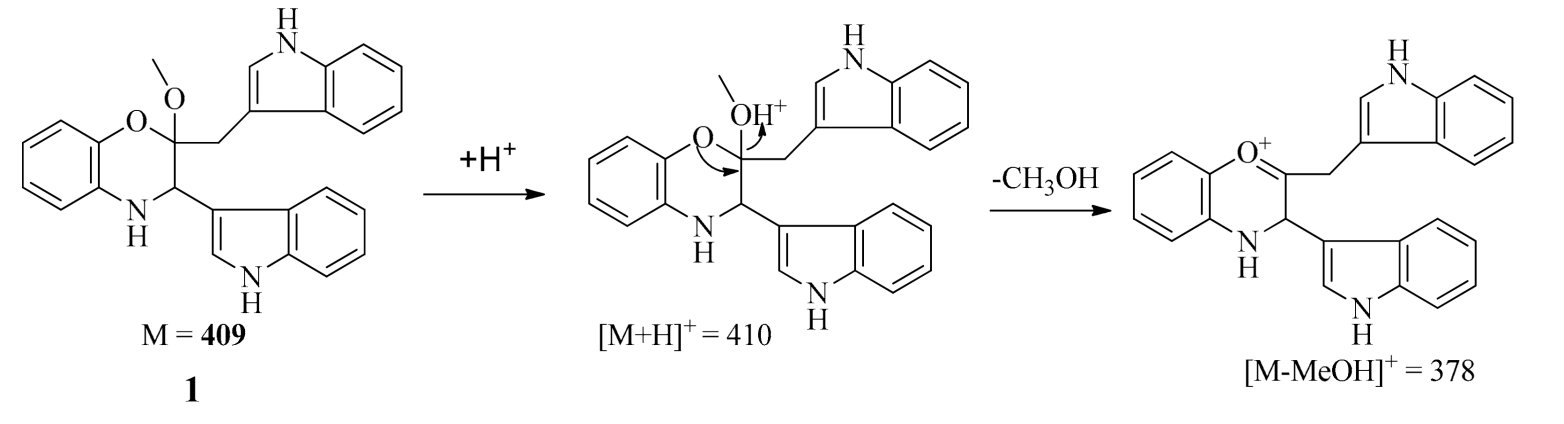
Possible formation for major fragment of **1** in ESI-TOF-MS (positive) and LC-MS (positive) spectra.

The positive-ion ESI-TOF-MS and LC-MS (positive) spectra of **1** both showed a major fragment ion peak at *m*/*z* 378 (the most intense peak), which may be formed through loss of a MeOH molecule as shown in Scheme 1. The formula of the major ionic fragment of **1** at *m*/*z* 378 was determined to be C_25_H_20_N_3_O^+^ on the basis of positive HR-ESI-MS (calcd. for C_25_H_20_N_3_O^+^ *m*/*z* 378.160089; found, 378.162728), which was in accordance with the proposed structure.

The recorded optical rotation (OR) for **1** was −6.8 (*c* = 0.055, MeOH). The observed OR was not zero, but the low measured specific rotation value might be due to chiral impurities as seen in the chiral HPLC chromatogram, or it might be a measurement artifact which cannot be avoided since the sample concentration was very low. This sample was further analyzed by HPLC on a chiral ODS-RH phase column (80% acetonitrile in 20% water over 40 min, 0.3 mL/min, 284 nm, 25 °C). Two UV absorption related peaks **1a** and **1b** with almost equal integral area (50:50) appeared in the HPLC profile (see Supplementary Materials), indicating that compound **1** is an almost 50:50 mixture of two enantiomers **1a** and **1b**, further confirming our speculation that compound **1** is a racemic mixture. The relative configurations of C-2 and C-3 in **1** were determined by 1D- and 2D-NOESY experiments. The NOESY correlation observed between H-2 and H-10 indicated that H-2 and H-10 should be located on the same side, which was further confirmed by the evident that there is no NOE correlation signals observed when irradiated H-2 and H-20 in 1D-NOE experiment, respectively. Finally, compound **1** was identified as (±)-2-(1*H*-indol-3-yl)-3-((1*H*-indol-3-yl)methyl)-3-methoxy-1,2-dihydro-2*H*-benzo[*b*][1,4] oxazine. Due to the limited sample amount available, we did not further separate this racemic mixture and determine the absolute configurations.

Two known indole trimers **2** and **3** were identified as trisindoline (**2**) [16] and 2,2-di(3-indolyl)-3-indolone (**3**) [17], respectively, based on the comparison of their spectroscopic data with those reported in the literature.

The antibacterial activities of compounds **1**–**3** against *Bacillus cereus* were investigated using the paper disk method. The results showed that compound **2** exhibited very strong antibacterial activity, with approximately 20 mm diameter growth inhibition at 10 ug/paper. This compound was first reported as a new antibiotic produced by a bacterium of *Vibrio* sp. isolated from a marine sponge [16], and subsequently reported as the gene specific product with cytotoxic activity by *E. coli* expressing the plant-derived oxygenase gene [18]. Compounds **1** and **3** exhibited no inhibitory effect against *B. cereus*. Thus, we predicted that compound **2** would be a gene-specific metabolite in clone PDC115. 

**Figure 3:**
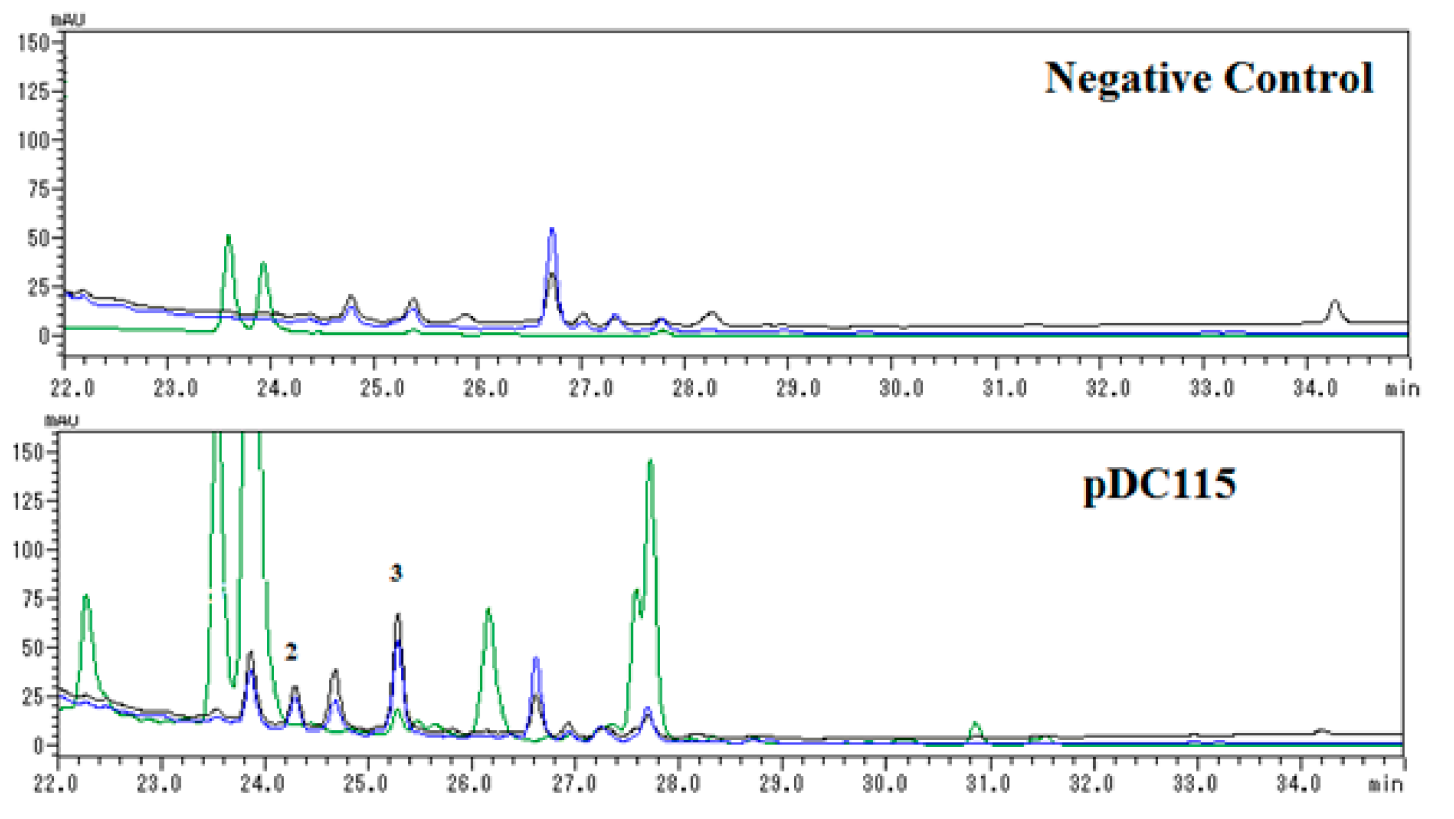
HPLC profiles of 100% CH_3_OH portions of negative control and pDC115 separated by ODS chromatography. Each sample was prepared to be 10 mg/mL and 5 μL volume was injected for analysis. Elution was monitored by UV absorptions at three representative wavelengths including 254 (black line), 284 (blue line), and 405 (green) nm.

Further analysis of HPLC profiles (Figure 3) of negative control (*Escherichia coli* carrying a void vector) and clone PDC115 indicated that compound **2** was a newly induced compound, while the concentration of **3** was obviously enhanced in contrast to negative control. Unfortunately, new compound **1** was not detected in HPLC analysis of the negative control (*Escherichia coli* carrying a void vector) and clone PDC115, suggesting it might be formed during the isolation procedure.

**Scheme 2:**
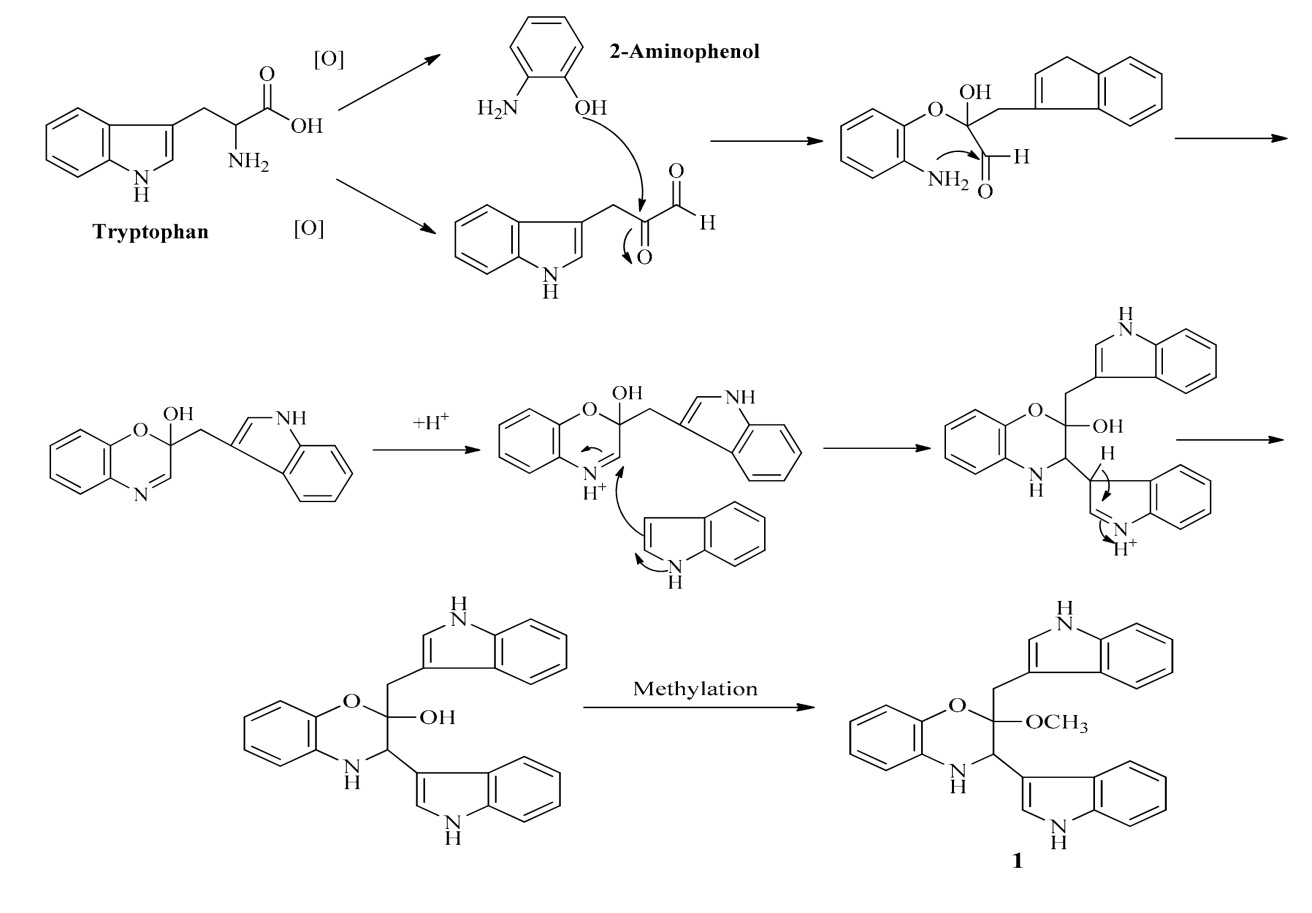
The proposed formation routine of **1** from tryptophan.

Thus, a hypothetical route of formation shown in Scheme 2 might be envisaged, leading from tryptophan to **1** via the unmethylated precursor which could be methylated during the isolation procedure involving methanol. In order to further confirm the proposed formation of **1**, we checked the crude extract by LC-MS, unfortunately, the proposed key intermediate (the desmethyl form of **1**) was not detected in the crude extract. 

## 3. Experimental Section 

### 3.1. General Experimental Procedures

1D-(including ^1^H- and ^13^C-NMR) and 2D-NMR (including COSY, HSQC, HMBC and NOESY) spectra were recorded on an ECX-500 spectrometer (JEOL, Tokyo, Japan) in DMSO-*d*_6_ with ^1^H and ^13^C nuclei observed at 500 and 125 MHz, respectively, using TMS as an internal standard, δ in ppm, *J* in Hz. HR-MS data were recorded on a Bruker Daltonics micro TOF-MS (Bruker Daltonics, Bremen, Germany) and JEOL JMS-200 MStation (FAB) instrumentz. LC-MS data were obtained from an 1100 series HPLC (Agilent, Santa Clara, USA)-microTOF mass spectrometer (Bruker Daltonics) system, using electrospray ionization with a Cadenza CD-C_18_ column (2.0 i.d. × 150 mm; Imtakt Co. Ltd. Tokyo, Japan). Optical rotations were measured on a DIP-1000 Digital Polarimeter (JASCO, Tokyo, Japan). The metagenomic library was constructed according to the protocol in the CopyControl™ Fosmid Libaray Production Kit with pCC1FOS^TM^ vector (Epicentre Technologies, Madison, WI, USA). DNA was extracted according to DNeasy^®^ Mini Kit (QIAGEN, Tokyo, Japan). Plasmid extraction was done according to Wizard^®^ Plus SV Minipreps DNA Purification System (Promega, Tokyo, Japan). DNA ligation was done according to DNA ligation kit ver. 2.1 (TaKaRa, Tokyo, Japan). *Bacillus cereus* was obtained from BioVector NTCC Inc. (Tokyo, Japan). The reagents of Tris, EDTA, SDS were supplied by Nacalai Tesque Inc. (Tokyo, Japan). Other reagents including NaCl, phenol, chloroform, ethanol, sodium acetate, isoamyl alcohol, urea, chloramphenicol, and glycerol were supplied by Wako Pure Chemical Industries, Ltd. (Tokyo, Japan).

### 3.2. Extraction of Metagenomic DNA from Discodermia calyx

The *Discodermia calyx* sample (ca. 100 g) was collected in May 2012 from a depth of approximately 10 m off Shikine-jima Island, an island administered by Japan located in the Philippine Sea, and identified by Professor Toshiyuki Wakimoto in the University of Tokyo. The sample was kept in a freezer at −80 °C. The voucher specimen (S11-001) was deposited at the Laboratory of Natural Products Chemistry, Graduate School of Pharmaceutical Sciences, the University of Tokyo. Before extracting metagenomic DNA, the frozen sponge was first broken into pieces under liquid nitrogen in a mortar, and then pulverized to a fine powder. Subsequently, small portions of the powder was dissolved in lysis buffer (50 mM Tris (pH 7.5), 50 mM EDTA, 350 mM NaCl, 8 M urea, 2% SDS with gently mixing and then incubated at 60 °C for 1 h. The crude extract DNA was washed three times with an equal volume of phenol–chloroform–isoamyl alcohol (25:24:1) and with chloroform–isoamyl alcohol (24:1), respectively. After precipitation with 2.5 volumes of ethanol and 1/10 volume of 3 M sodium acetate (pH 5.2) and centrifugation at 10,000× *g* for 20 min led to afford the DNA pellet, which was further washed with cold 70% ethanol, air-dried and then re-dissolved in TE buffer. To the metagenomic DNA solution, 0.5% SDS and 100 μg/mL proteinase K (TaKaRa) were added. After incubation at 50 °C for 1 h, the crude metagenomic DNA solution was washed again with phenolchloroform-isoamyl alcohol and chloroform-isoamyl alcohol as described above.

### 3.3. Purification of Metagenomic DNA and Library Construction

The crude metagenomic DNA was size-fractionated by agarose gel electrophoresis (0.8% low melting point agarose gel (TaKaRa), 50 V for 18 h). The DNA fragments larger than 35 kbp were recovered from the gel by GELare (Epicentre Technologies) according to the protocol in the CopyControl™ Fosmid Libaray Production Kit (Epicentre Technologies). The purified DNA was blunt-ended with an End-It DNA End-Repair Kit (Epicentre Technologies), and then ligated into the pCC1FOS fosmid vector (Epicentre Technologies). This vector was subjected to a packaging reaction with a MaxPlax Lambda Packaging Extract (Epicentre Technologies). The packaged vector was used to transfect *Escherichia coli* EPI300-95 T1R (Epicentre Technologies) and the cells were placed in the LB agar containing chloramphenicol (12.5 μg/mL) as a selection marker, according to the manufacturer’s protocol.

### 3.4. Antibacterial Screening

Almost 100 colonies on each plate were grown on LB agar medium containing chloramphenicol (15 ug/mL final concentration), at 30 °C for 2 days. Subsequently, 0.5% LB soft agar medium containing a *Bacillus cereus* culture solution was poured onto each agar plate, which was cultivated for 12 h at 37 °C. After the cultivation, the inhibition halo around each colony was monitored. When a positive clone was detected, it was picked up, inoculated into LB medium containing chloramphenicol, and maintained as a glycerol stock at −80 °C [13].

### 3.5. Extraction and Isolation

The marine sponge *Discodermia calyx* metagenomic fosmid library was constructed according to the CopyControl^TM^ Fosmid Libaray Production Kit (Epicentre Technologies), which contains 250,000 fosmid clones with an average DNA insert size of 40 kb. One positive clone with antibacterial activity against *Bacillus cereus*, designated pDC115, was found from the library based on functional screening. Thus, the positive clone pDC115 was selected for large-scale cultivation and detailed chemical analysis. The clone pDC115 was firstly pre-cultured overnight in LB broth at 37 °C, and then 10 mL of culture liquid were transferred as seed into each 2500 mL Erlenmeyer flask containing 1500 mL of LB medium. After three days of cultivation in LB broth (36 L) at 30 °C on rotary shakers at 120 rpm. The medium was subjected to solid phase extraction using Diaion HP-20 resins (250–850 μm, Mitsubishi Chemical Corporation, Tokyo, Japan). The methanol extract was subjected to ODS column chromatography (Cosmosil 75C18-PREP, 75 μm, Nacalai Tesque) eluted with a stepwise gradient system from water to methanol (0, 25%, 50%, 75%, and 100% methanol (*v/v*)) to afford five fractions *Fr. 1-5*. The activity screening indicated that *Fr. 4* eluted with 75% methanol is the active fraction. Further bioassay-guided fractionation of *Fr. 4* by repeated Sephadex LH-20 (MeOH) and semipreparative reversed-phase HPLC (Shimadzu, LC-20AD and SPD-20A Prominence Diode Array Detector) using Cosmosil 5C18-PAQ Waters (10 mm × 250 mm, Nacalai Tesque) with a mixture of H_2_O and CH_3_CN, both containing 0.1% acetic acid at a flow rate of 2.5 mL/min afforded **1** (1.2 mg, *t*_R_ = 14.5 min, 46% CH_3_CN in H_2_O), **2** (1.5 mg, *t*_R_ = 26.5 min, 84% CH_3_CN in H_2_O) and **3** (1.2 mg, *t*_R_ = 28.5 min, 90% CH_3_CN in H_2_O).

*(±)-2-(1H-Indol-3-yl)-3-((1H-indol-3-yl)methyl)-3-methoxy-1,2-dihydro-2H-benzo[b][1,4] oxazine* (**1**): colorless amorphous powder; ${\left[\alpha \right]}_{D}^{24}$ = −6.8º (*c* 0.055, MeOH); ^1^H- and ^13^C-NMR data: Table 1; HR-ESI-MS at *m*/*z* 410.1887 (calcd. for C_26_H_24_N_3_O_2_, *m*/*z* 410.1863).

*Trisindoline* (**2**): colorless amorphous powder; ^1^H-NMR (DMSO-*d*_6_): two tri-substituted indole-ring proton signals at δ_H_ 7.33 (2H, d), 7.27 (2H, m), 7.03 (2H, t), 6.90 (2H, s) and 6.80 (2H, t), and one di-substituted indole proton signals at δ_H_ 7.27 (1H, m), 7.25 (1H, m), 7.05 (1H, d) and 6.98 (1H, t); HR-ESI-MS at *m*/*z* 364.1470 (calcd. for C_24_H_18_N_3_O, 364.1444).

*2,2-Di(3-Indolyl)-3-indolone* (**3**): colorless amorphous powder; ^1^H-NMR (DMSO-*d*_6_): two trisubstituted indole-ring proton signals at δ_H_ 10.95 (2H, s, NH), 7.31 (2H, d), 7.29 (2H, m), 6.99 (2H, t), 6.80 (2H, s) and 6.80 (2H, t), and one di-substituted indole proton signals at δ_H_ 8.10 (1H, s, NH), 7.47 (1H, d), 7.44 (1H, t), 6.91 (1H, d) and 6.70 (1H, t); HR-ESI-MS at *m*/*z* 364.1442 (calcd. for C_24_H_18_N_3_O, 364.1444).

### 3.6. Antibacterial Assay

The antibacterial activity of compounds **1**–**3** was determined according to the protocol reported in [13]. Screening plates containing *Bacillus cereus* were prepared with LB agar medium. All isolated compounds were dissolved in DMSO to a final concentration of 1 mg/mL. Subsequently, 10, 25, 50, and 100 uL were applied to 0.7 mm paper disks (Advantech, Tokyo, Japan). The disks were placed on the prepared plates and incubated at 30 °C for 16 h. Inhibition was scored visually, and zones of inhibition were reported as the diameter of the clear zone in millimeters. The assay was conducted in duplicate trials.

## 4. Conclusions

The present work reports three indole derivatives **1**–**3**, including a novel benzoxazine-indole hybrid **1** from *Escherichia coli* harboring the marine sponge *Discodermia calyx* metagenomic DNA. HPLC analyses revealed that compound **2** is a newly induced metabolite, and the concentration of **3** was obviously enhanced in comparison with the negative control. Compound **1** as racemic mixture was not detected in the liquid culture, which leads to the speculation that the formation of **1** could be due to non-enzymatic processes during the isolation procedure. Biological study indicated that only compound **2** displayed significant antibacterial activity against *Bacillus cereus*, with approximately 20 mm diameter growth inhibition at 10 µg/paper, suggesting that this compound might be a gene-specific metabolite in clone PDC115. 

## Figures and Tables

**Table 1:** ^1^H- (500 MHz) and ^13^C-NMR (125 MHz) data for **1** in DMSO-*d*_6_ (δ in ppm, *J* in Hz).

No.	δ_H_	δ_C_	No.	δ_H_	δ_C_
1	5.69 (1H, s)		16	7.43 (1H, d, 8.0)	110.9 (d)
2	4.46 (1H, s)	51.2 (d)	17	6.92 (1H, overlap)	118.2 (d)
3		97.7 (s)	18	7.07 (1H, overlap)	120.7 (d)
4		132.9 (s)	19	7.39 (1H, d, 8.5)	123.9 (d)
5	6.57 (1H, d, 8.0)	113.8 (d)	20a	3.46 (1H, overlap)	28.7 (t)
6	6.66 (1H, t, 7.9)	119.9 (d)	20b	2.89 (1H, d, 15.5)	
7	6.51 (1H, t, 7.5)	116.3 (d)	21		107.5 (s)
8	6.81 (1H, brs)	115.8 (d)	22	6.81 (1H, brs)	124.1 (d)
9		138.8 (s)	23	10.83 (H, s)	
10	3.17 (3H, s)	48.3 (q)	24		133.9 (s)
11		110.4 (s)	25		126.6 (s)
12	7.30 (1H, brs)	125.1 (d)	26	7.57 (1H, d, 8.5)	119.2 (d)
13	11.09 (1H, s)		27	6.92 (1H, overlap)	118.2 (d)
14		133.9 (s)	28	7.02 (1H, overlap)	120.2 (d)
15		125.6 (s)	29	7.30 (1H, brs)	110.9 (d)

## Supplementary Materials

The original spectra of NMR, LC-MS, chiral HPLC analysis, and HR-ESI-MS data for compound **1** are available as Supplementary Materials.
