# Supplementary material for: Indole Derivatives Produced by the Metagenome Genes of the Escherichia coli-Harboring Marine Sponge Discodermia calyx
Source: Molecules. 2017 Apr 25;22(5):681. doi: 10.3390/molecules22050681 (PMC6154727; doi:10.3390/molecules22050681)
Supplement: Supplementary file 1 [file molecules-22-00681-s001.pdf]

Supporting Information

Indole Derivatives Produced by the Metagenome Genes of the Escherichia coli-Harboring Marine Sponge Discodermia calyx

Feng-Lou Liu[1], and Xiao-Long Yang[2,*]
[1] Agricultural college,Ningxia University, Yinchuan 750021, Ningxia, China; liufenglou@nxu.edu.cn
[2] Innovative Drug Research Centre (IDRC), School of Pharmaceutical Sciences, Huxi Campus, Chongqing University, Chongqing 401331, China

List of Supporting Information

S1. Comparative data of indole trimer production in the clones… ……………………………………………………………………….......3

S2. LC-MS data of compound **1**.................……………………………….. ………………………………………………………………….........3

S3. Chiral HPLC analysis of compound **1**........……………..………….. ………...3

S4. HR-ESI-MS data of compound **1**………..…..…………………….. ……………………………………………………………… 4

S5. NMR spectrum of **1** ………………….……………….….. …………………………………………………………………………...………5

S1. Comparative data of indole trimmer production in the clones

The culture of negative control (means *E. coli* carrying void vector) (1.5 L) and pDC115 (1.5 L) were subjected to solid phase extraction using HP-20 resin, respectively. The methanol extracts were further separated by ODS column chromatography (Cosmosil 75C18-PREP, Nacalai Tesque) eluted with a stepwise gradient system from water to methanol to afford four fractions. The 100% methanol fractions from pDC115 and NC cultures were subjected to DAD-HPLC analysis. HPLC analysis was performed on ODS column (Cosmosil 5C18 PAQ waters, 4.6 x 250 mm) with a mixture of H_2O and MeCN, both containing 0.05% trifluoroacetic acid: 0–30 min, 5-100% MeCN; 30–50 min, 100% MeCN; 50–55 min, 100-5% MeCN; 55–60 min, 5% MeCN. Flow rate: 0.8 mL/min. DAD profile were measured with the Shimadzu HPLC System: LC-20AD and SPD-20A Prominence Diode Array Detector.

S2. LC-MS data of compound 1

LC-MS (Agilent 1100 series-Bruker Esquire 4000, positive ESI.) analysis was performed on ODS column (TSK-Gel ODS-80Ts, 4.6 x 150 mm) with a mixture of H_2O and MeOH. Flow rate: 0.2mL/min. Detection wavelength: 405 nm.

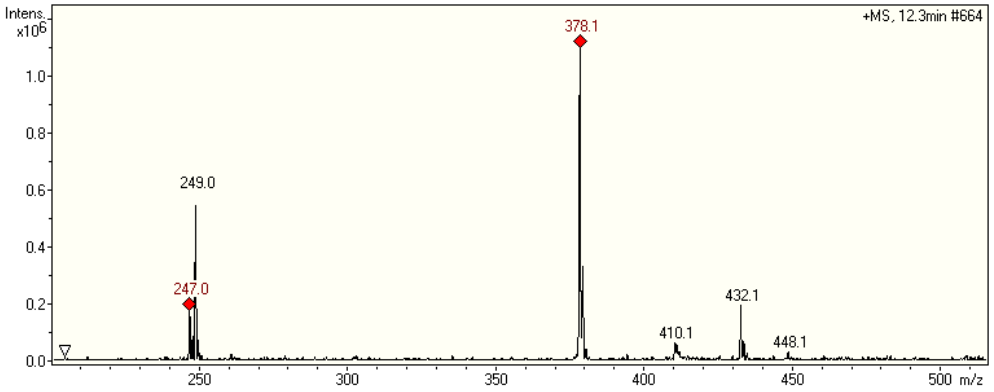

Figure S2. LC-MS (pos.) data of compound **1**

S3. Chiral HPLC analysis of compound 1

Chiral HPLC analysis was performed on ODS-RH column (5 *u*m, 150 x 4.6 mm, Daicel, city, Japan) with 80% MeCN in H_2O. Flow rate: 0.3mL/min, column pressure: 3 MPa. DAD profile were measured with the Shimadzu HPLC System: LC-20AD and SPD-20A Prominence Diode Array Detector.

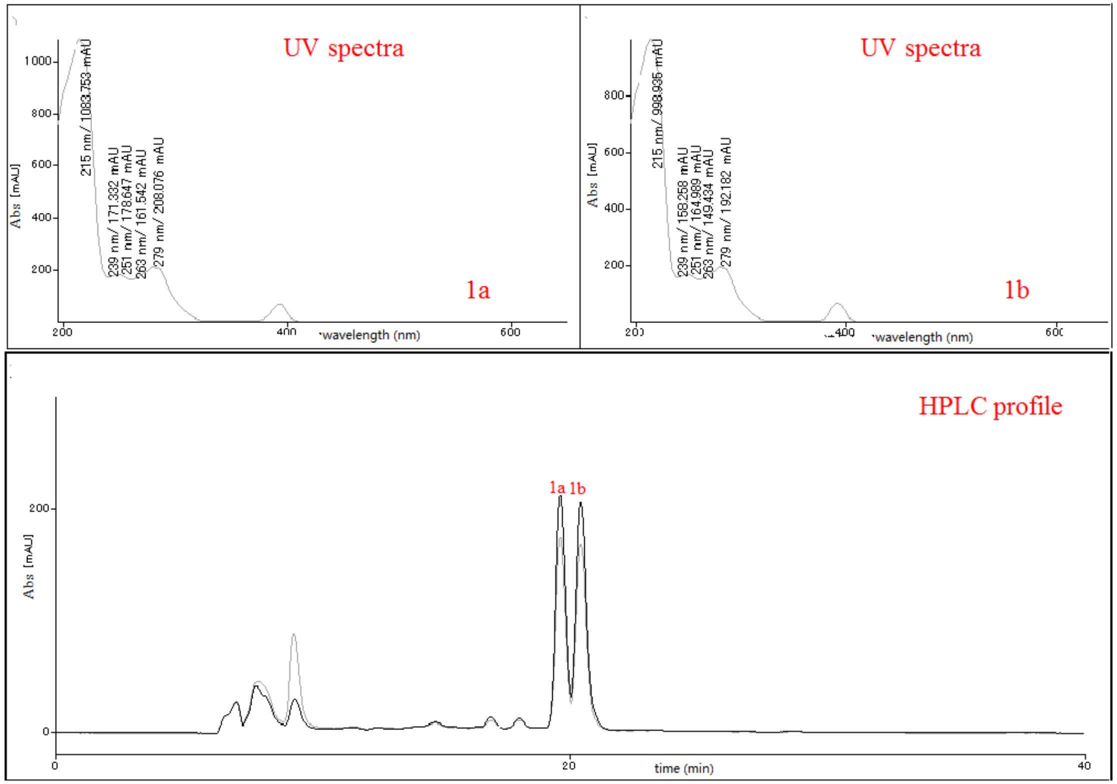

Figure S3. Chiral HPLC analysis of compound **1** and UV spectra for peaks **1a** and **1b**

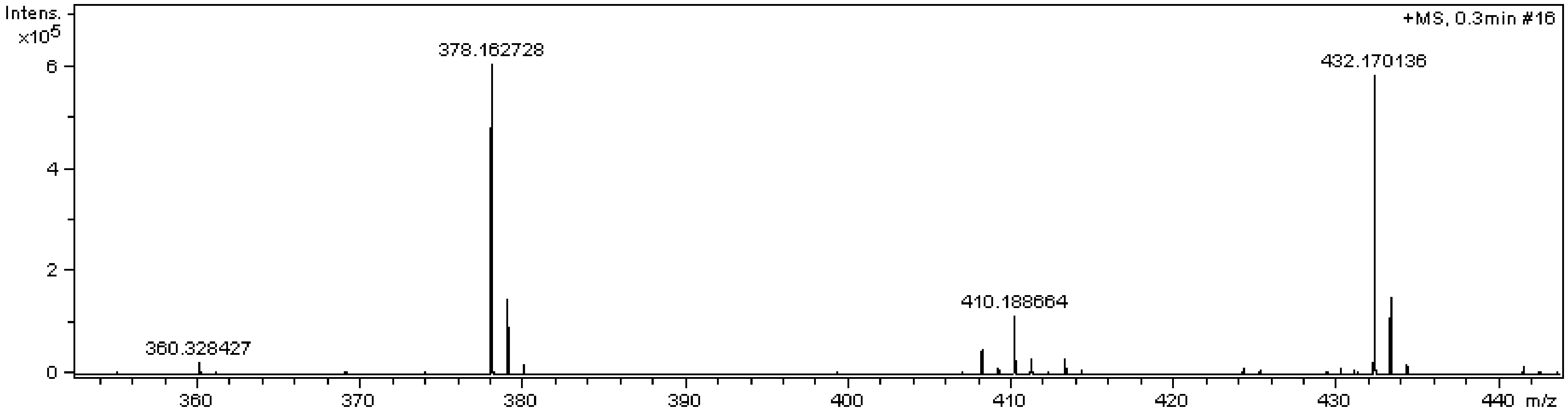

Figure S4. HR-ESI-MS (positive model) of compound **1**

S5. NMR spectrum of compound 1

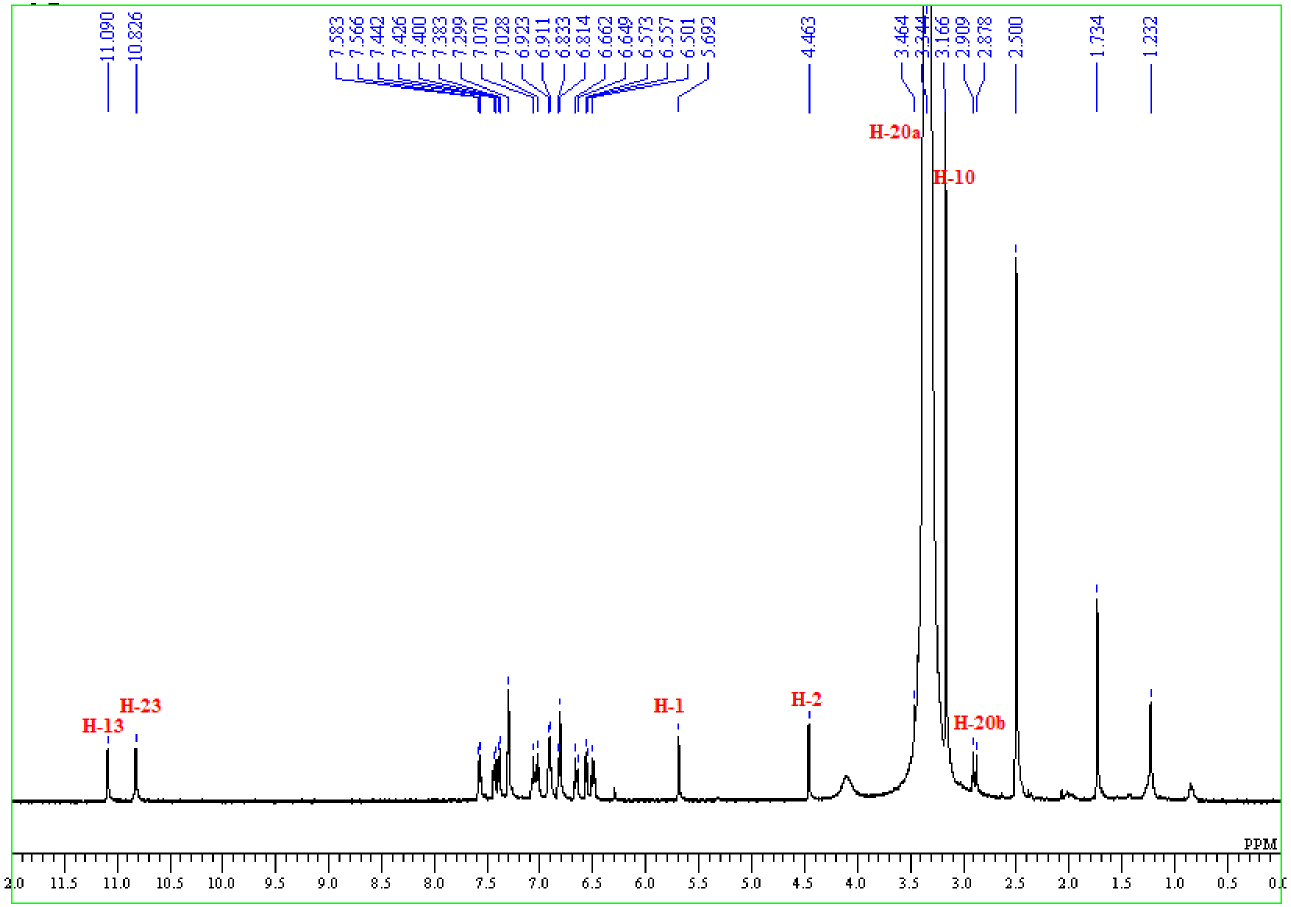

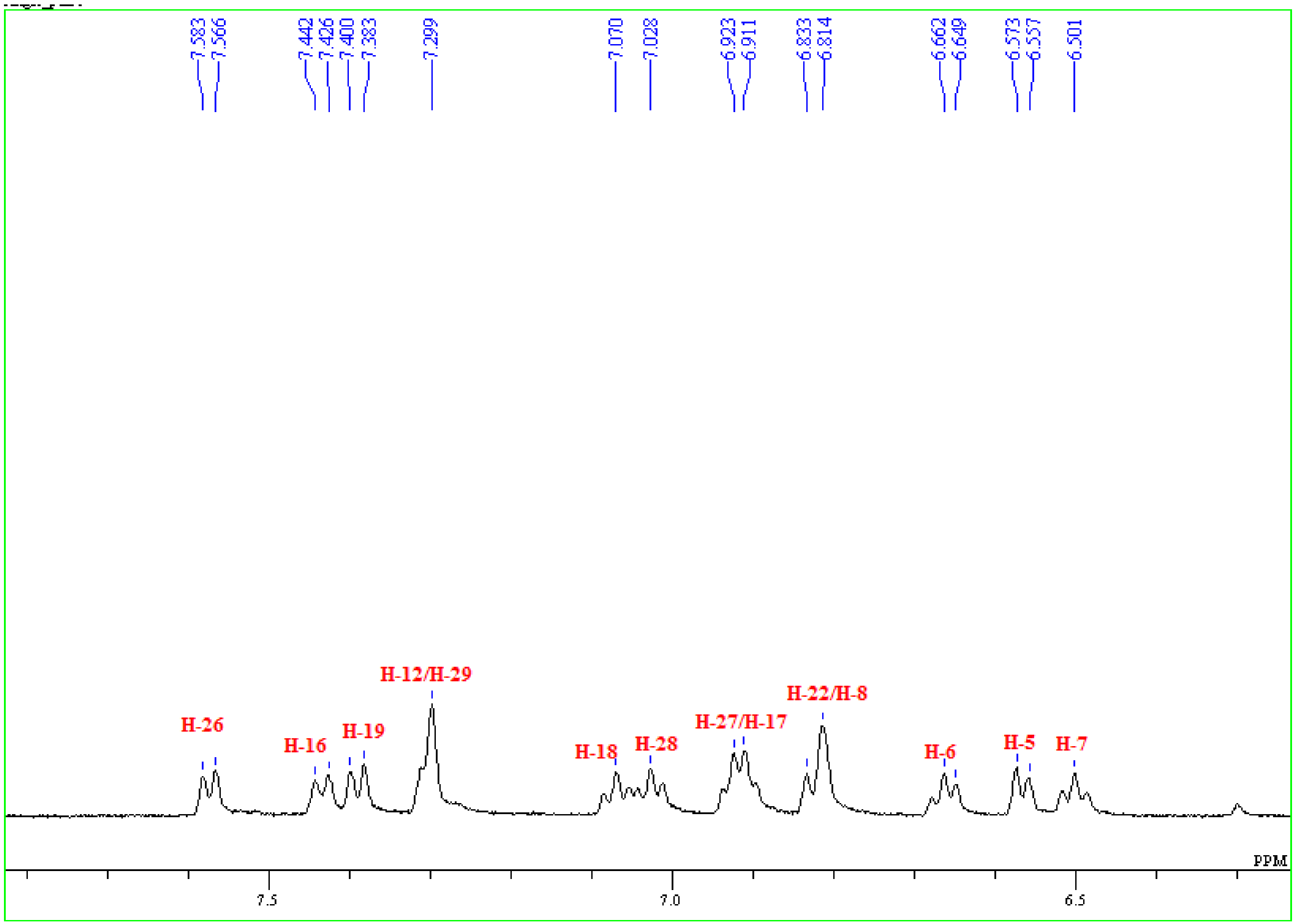

Figure S5-1. ^{1}H-NMR spectrum of **1** (500 MHz, DMSO-d_6)

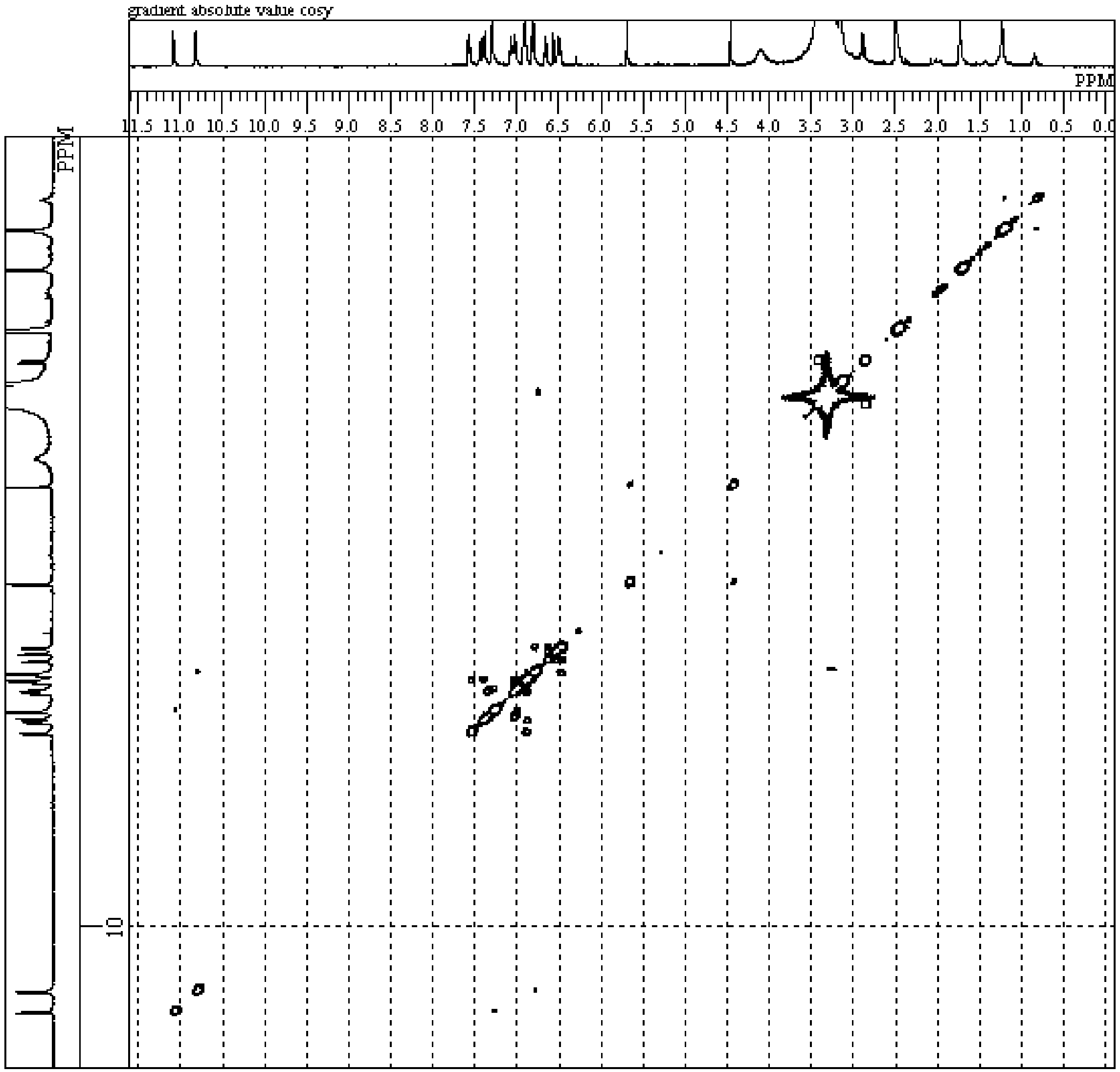

gradient absolute value cosy
PPM
11.5 11.0 10.5 10.0 9.5 9.0 8.5 8.0 7.5 7.0 6.5 6.0 5.5 5.0 4.5 4.0 3.5 3.0 2.5 2.0 1.5 1.0 0.5 0.0
PPM
10

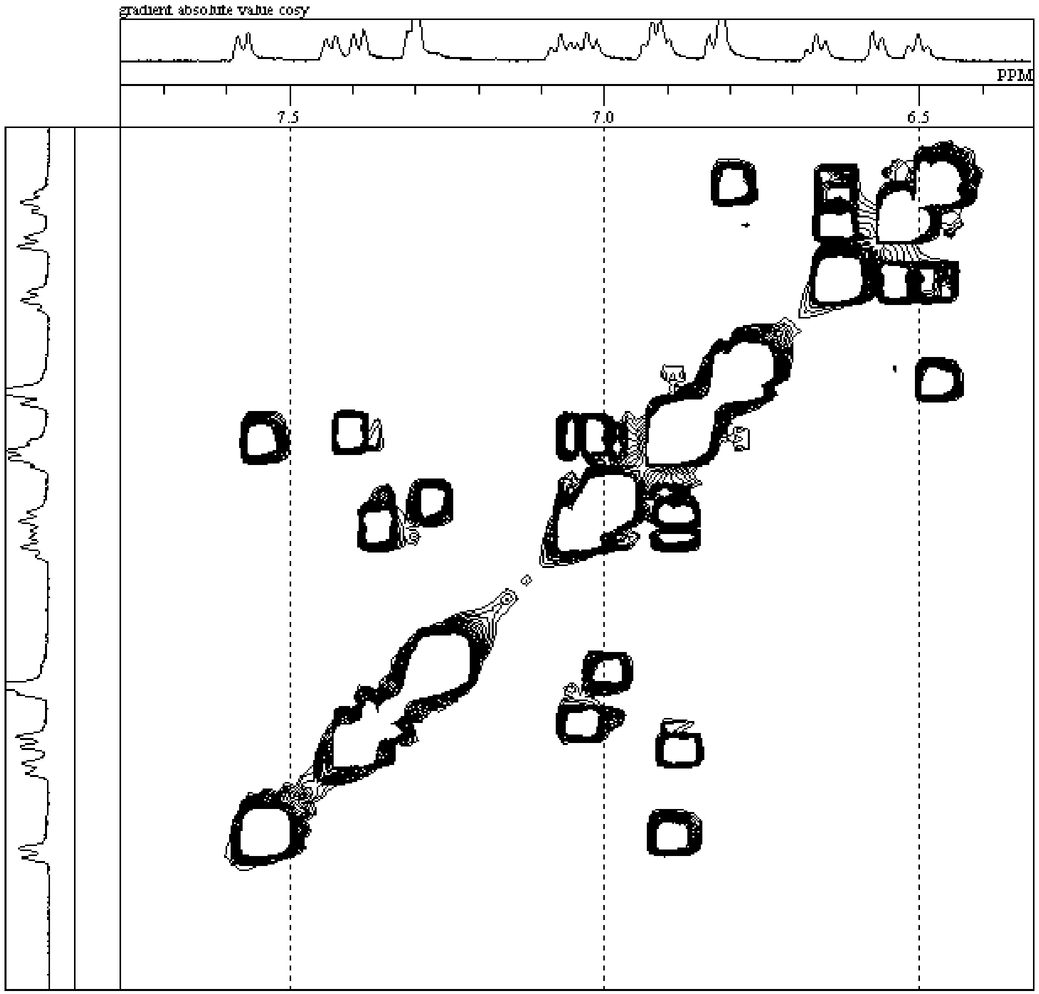

Figure S5-2. $^{1}H,^{1}H$-COSY spectrum of **1**

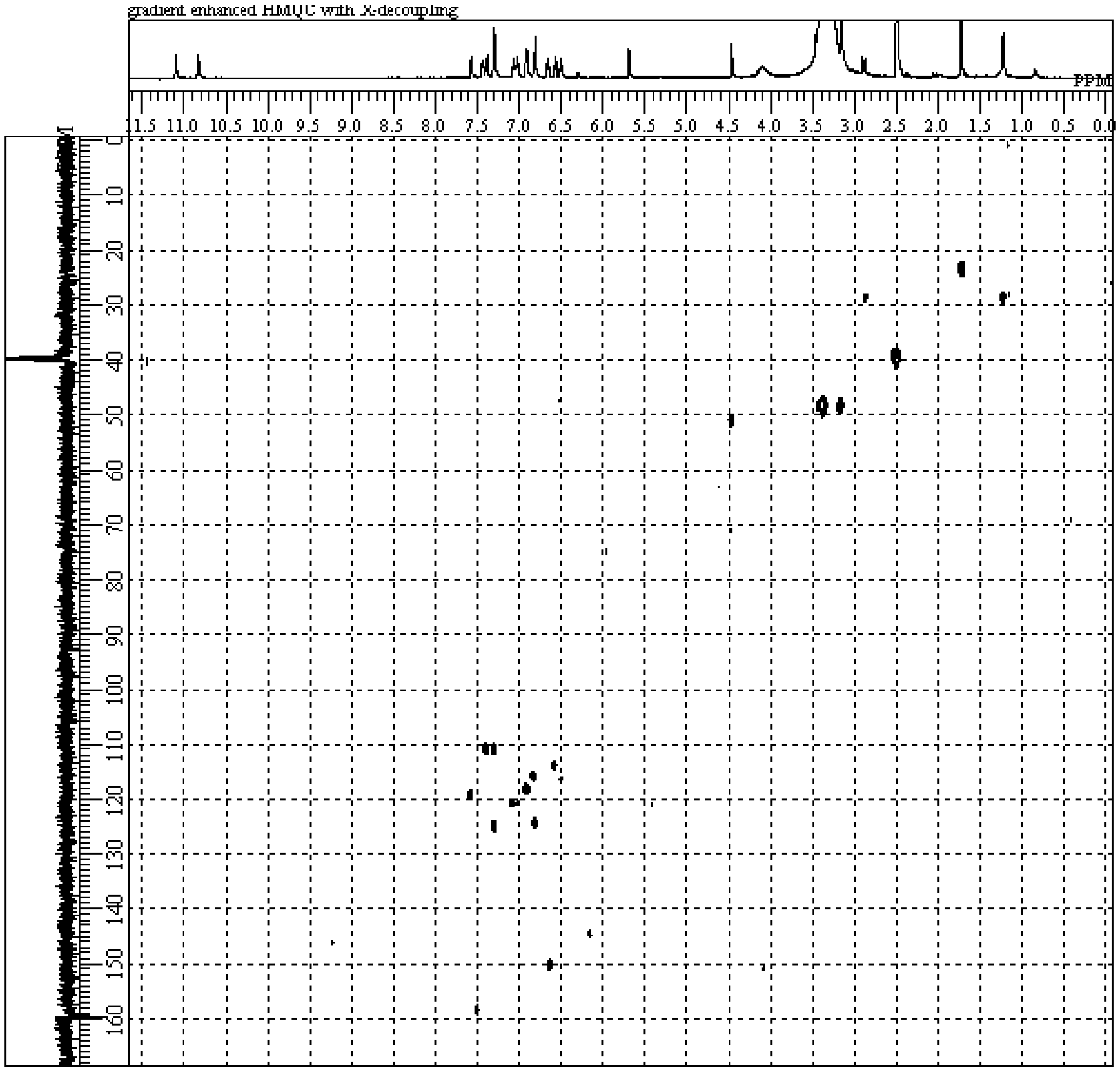
gradient enhanced HMQC with X-decoupling
PPM

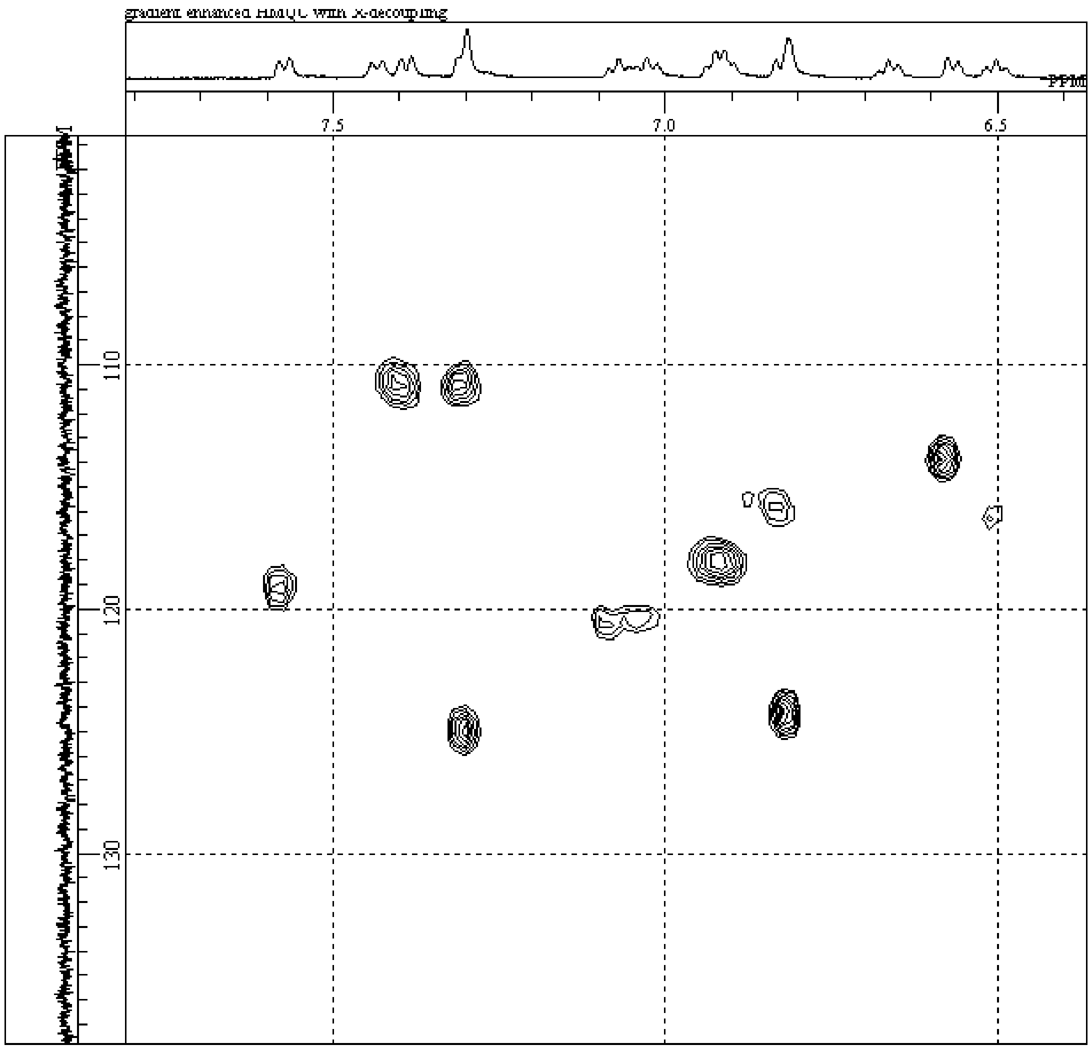

gradient enhanced HMQC with X-decoupling
PPM
7.5
7.0
6.5
110
120
130

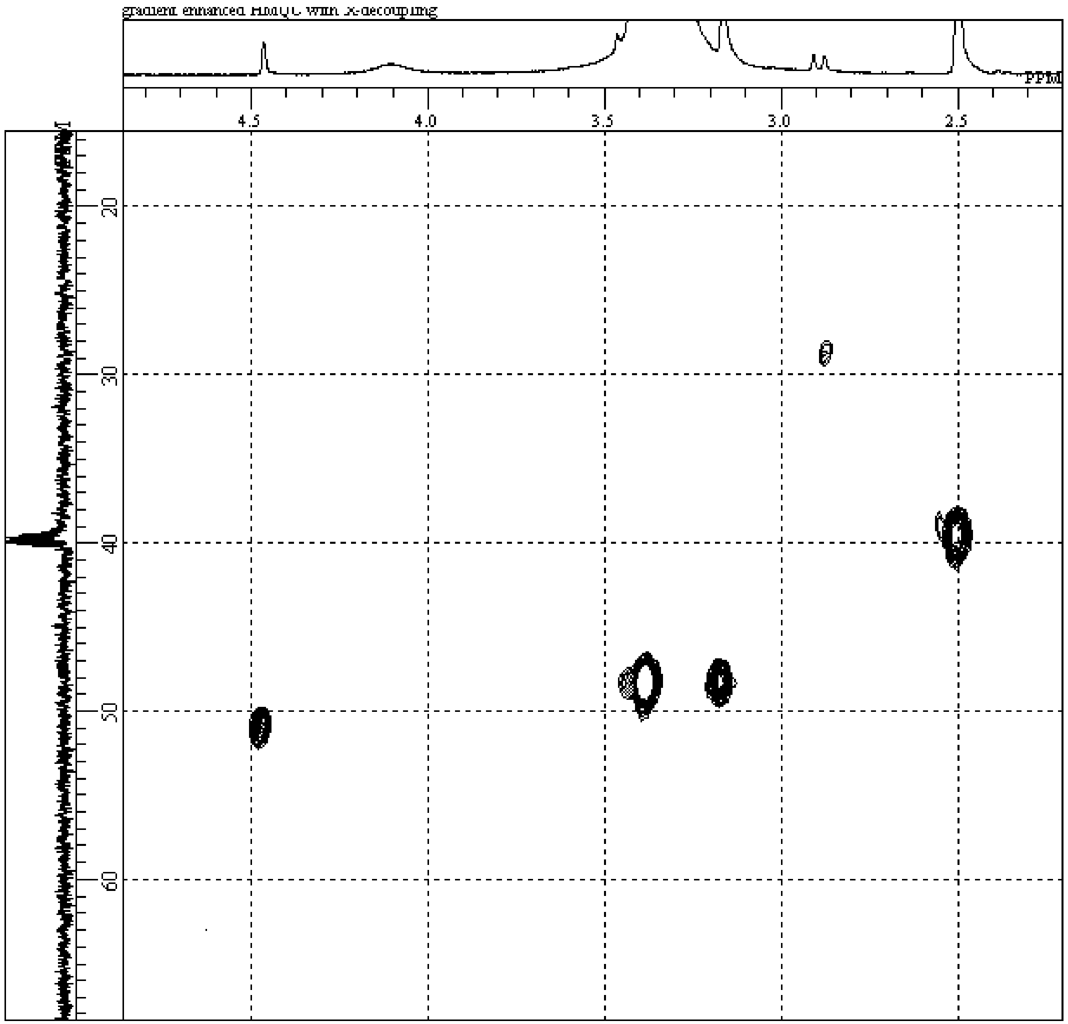

Figure S5-3. HSQC spectrum of **1**

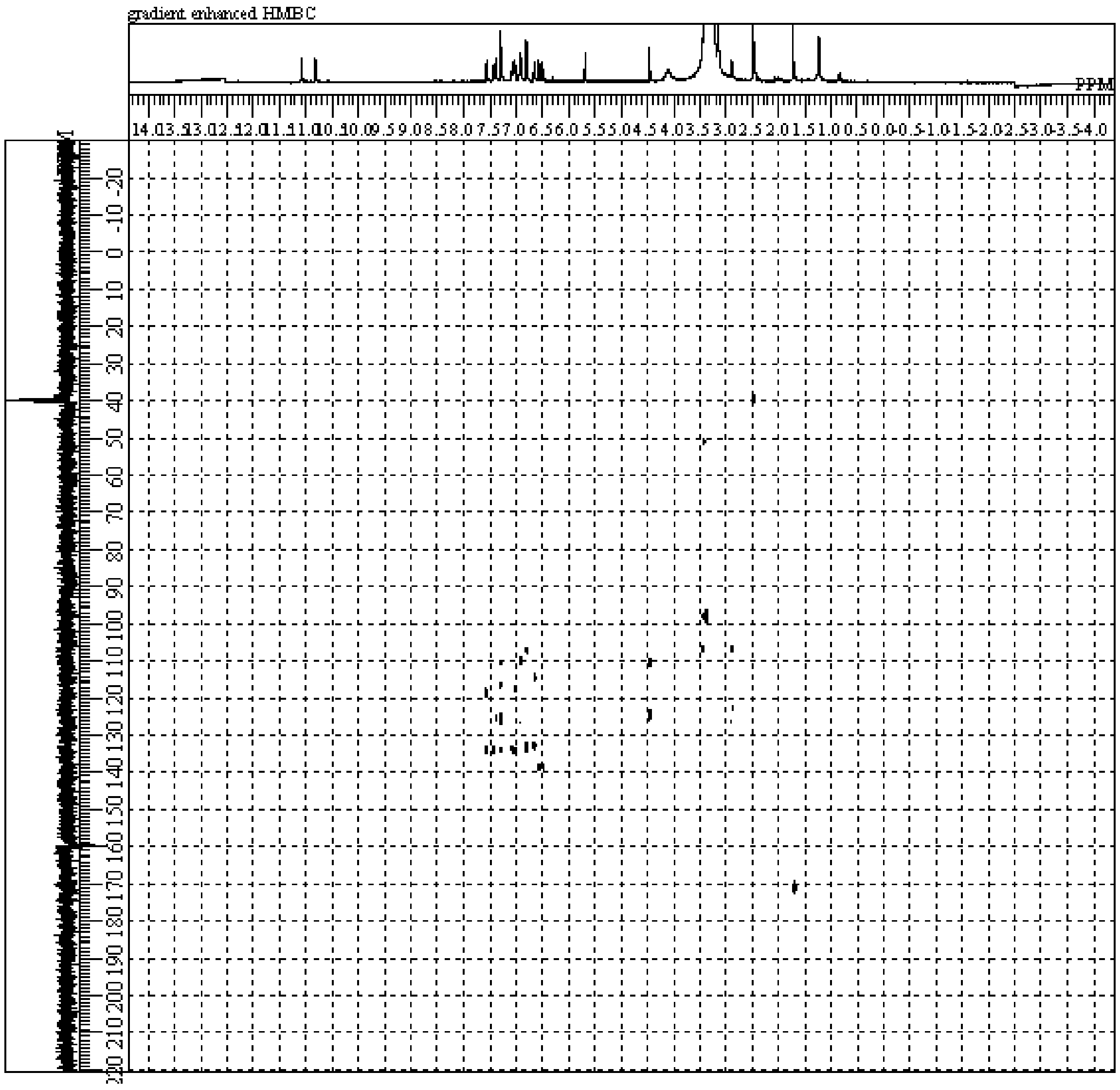
gradient enhanced HMBC
PPM

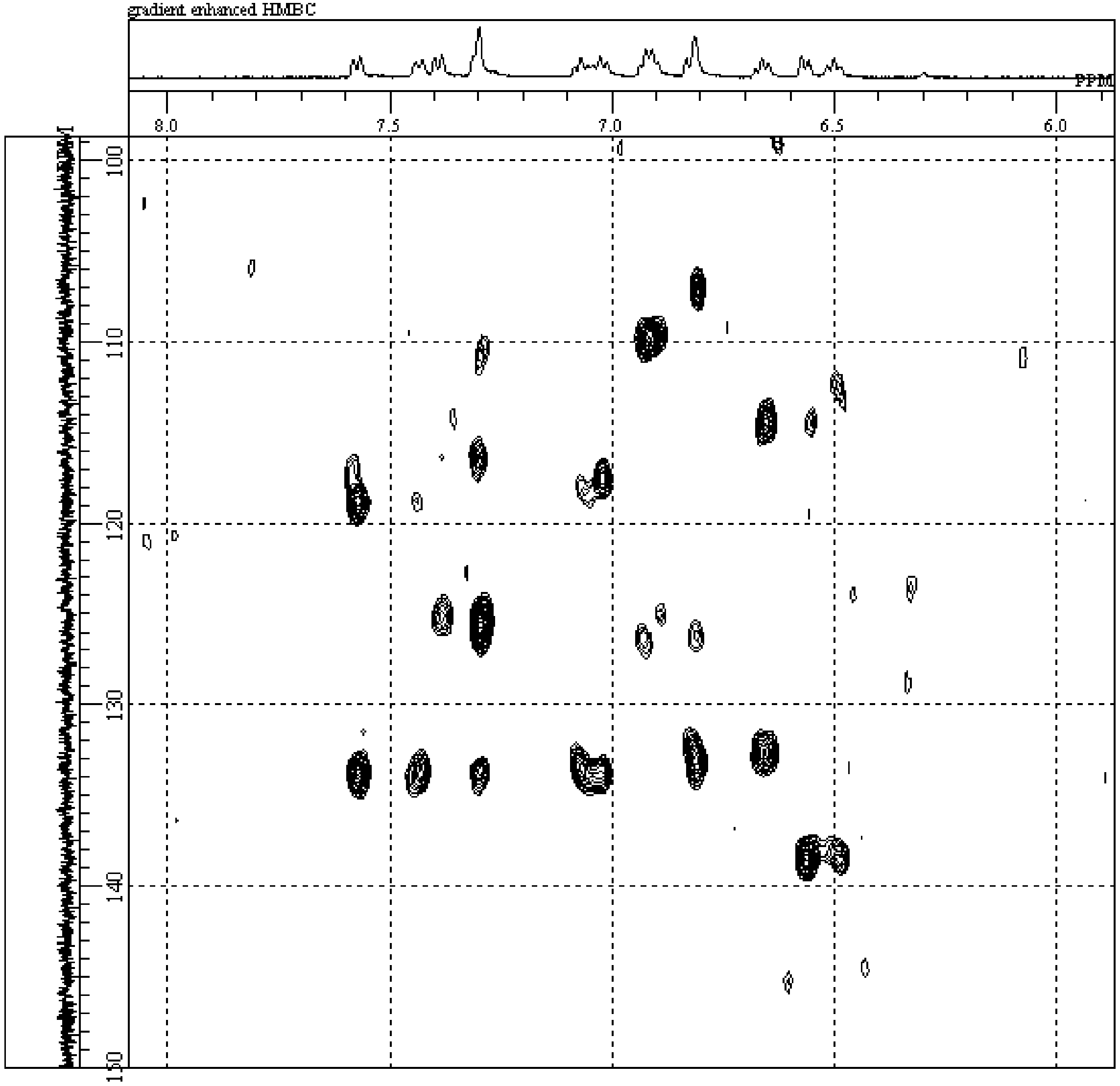

gradient enhanced HMBC
PPM
8.0
7.5
7.0
6.5
6.0
100
110
120
130
140
150

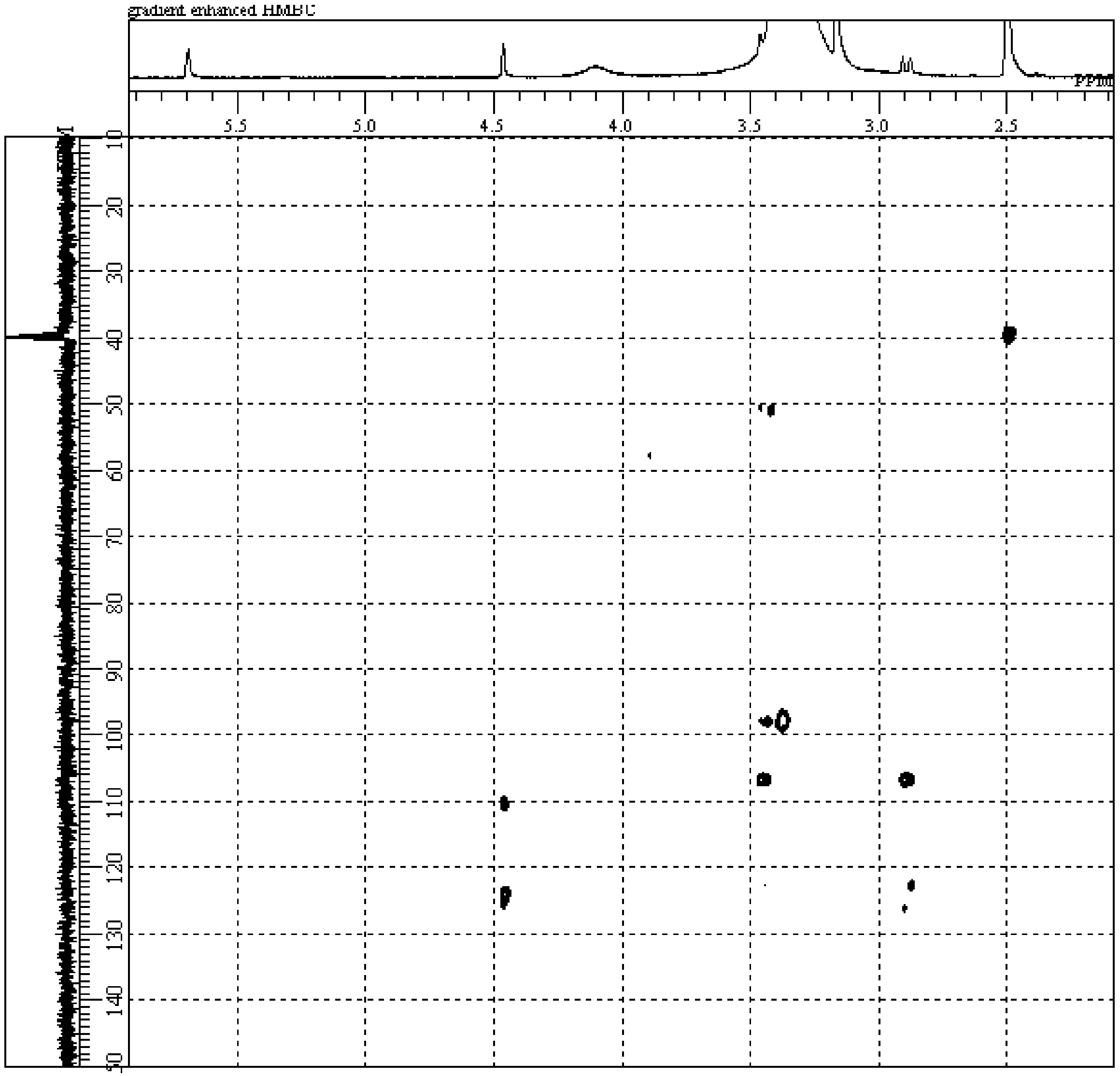
gradient enhanced HMBC
PPM
5.5
5.0
4.5
4.0
3.5
3.0
2.5
10
20
30
40
50
60
70
80
90
100
110
120
130
140
150

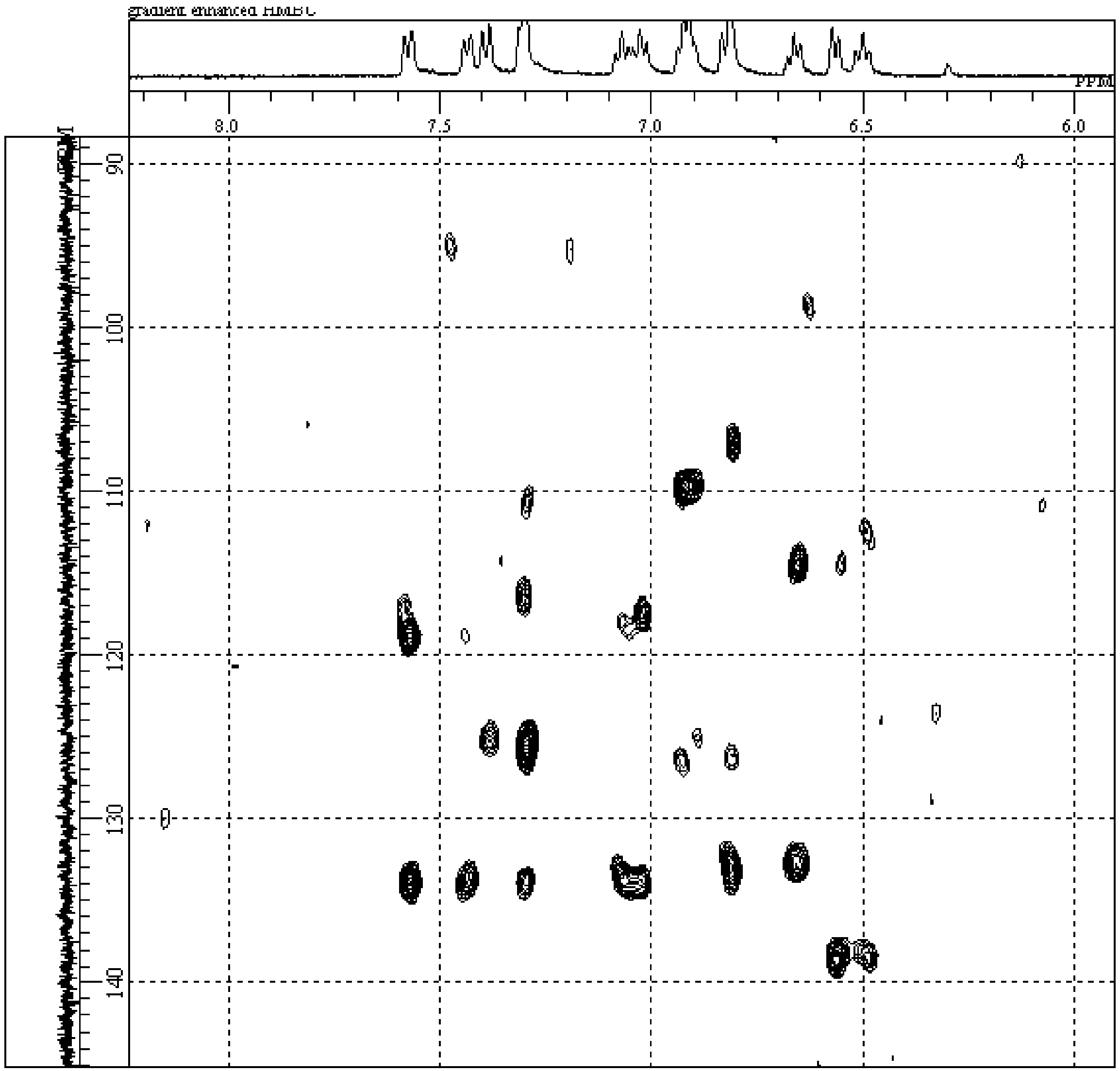

gradient enhanced HMBC
PPM
8.0
7.5
7.0
6.5
6.0
PPM
90
100
110
120
130
140

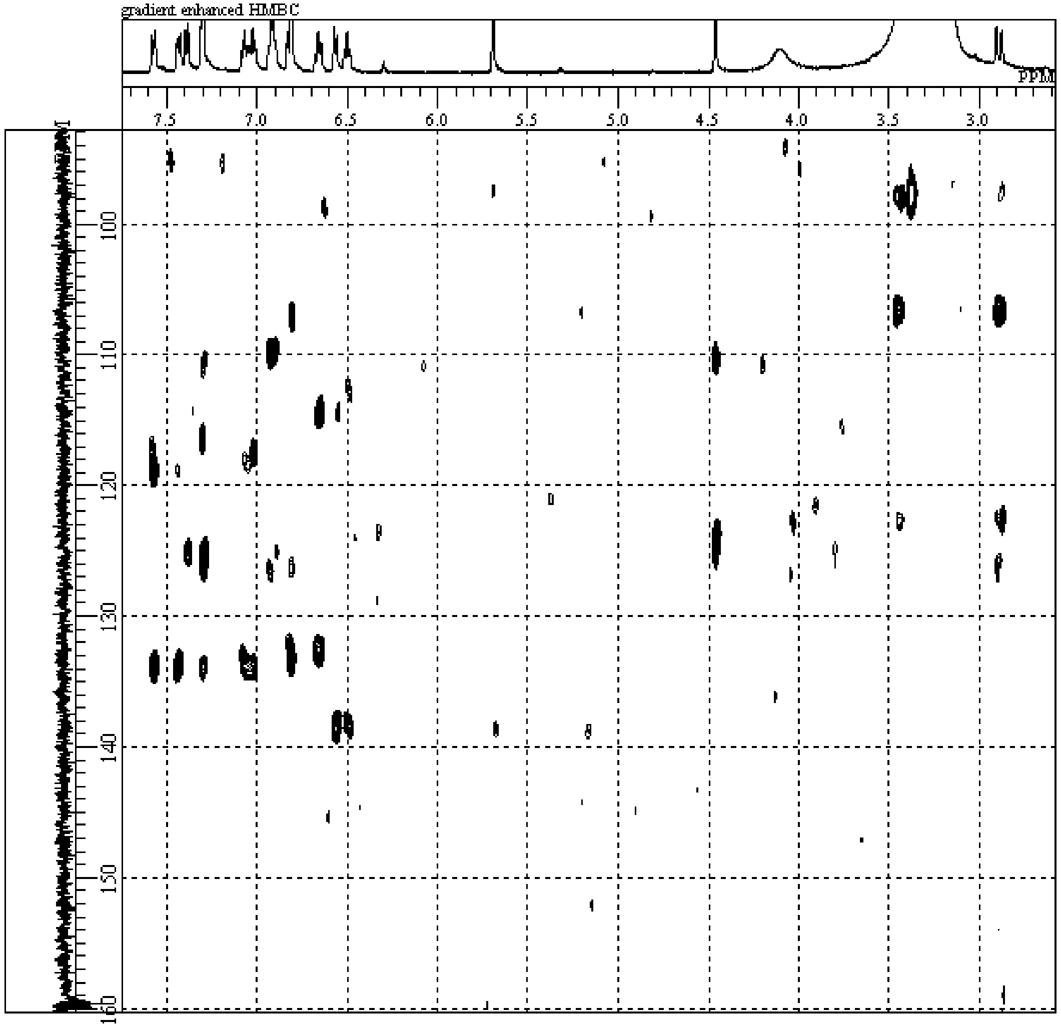

Figure S5-4. HMBC spectrum of **1**
